# Additive manufacturing of prosthetic devices: A scoping review of clinical outcomes

**DOI:** 10.33137/cpoj.v9i2.47836

**Published:** 2026-10-09

**Authors:** C Bhatla, M Ho, C Devion, R.Z Gao, A.L Mayo

**Affiliations:** 1. Division of Physical Medicine and Rehabilitation, Department of Medicine, Temerty Faculty of Medicine, University of Toronto, Toronto, Ontario, Canada.; 2. Michael G. DeGroote School of Medicine, McMaster University, Hamilton, Ontario, Canada.; 3. Library Services, Sunnybrook Health Sciences Centre, Toronto, Ontario, Canada.; 4. Sunnybrook Research Institute, Sunnybrook Health Sciences Centre, Toronto, Ontario, Canada.

**Keywords:** Additive Manufacturing, Three-Dimensional Printing, Artificial Limbs, Prosthetic Devices, Limb Loss, Scoping Review

## Abstract

**BACKGROUND:**

Additive manufacturing (3D printing) has been proposed as a means of improving customization, accessibility, and production efficiency in prosthetic fabrication. However, clinical outcomes remain incompletely characterized, with existing evidence limited by heterogeneous study designs, small samples, and inconsistent outcome reporting.

**OBJECTIVE:**

To conduct a scoping review to map the available evidence on clinical outcomes of 3D-printed prosthetic devices in individuals with limb loss or congenital limb difference.

**METHODOLOGY:**

JBI (formerly Joanna Briggs Institute) methodology and Preferred Reporting Items for Systematic Reviews and Meta-Analyses extension for Scoping Reviews (PRISMA-ScR) guidelines were followed. MEDLINE, Embase, CINAHL, CENTRAL, and Web of Science were searched from inception to December 31, 2025. Studies were eligible if they reported clinical outcomes, assessed using an identifiable method of measurement, for a functional limb prosthesis directly fabricated using additive manufacturing.

**RESULTS:**

Of 1,396 records screened, 42 studies were included. Thirty-one (73.8%) were case reports or case series; no randomized controlled trials were identified. Thirty-two studies (76.2%) involved upper-limb prostheses and ten (23.8%) involved transtibial devices. Fused deposition modeling (FDM) was the most reported additive manufacturing method (76.2%). In upper-limb studies without a comparator, participants frequently reported improved gross motor function compared with using no prosthesis, while fine motor performance and device durability were common limitations. Where a conventional device was used as a comparator, findings were mixed rather than uniformly favoring either fabrication approach. In lower-limb studies, gait and patient-reported outcomes were generally comparable between 3D-printed and conventionally fabricated devices.

**CONCLUSION:**

The literature describes 3D-printed prostheses being fabricated, fitted, and used across a range of clinical contexts, including for customized and activity-specific designs, but the evidence base remains limited. Future work should prioritize larger comparative studies, longer follow-up, and more standardized outcome assessment to better define the role of additive manufacturing in prosthetic care.

## INTRODUCTION

Prosthetic devices aim to restore mobility, function, and quality of life for individuals with limb loss or limb difference.^1^ They replace missing limbs, helping users regain independence in activities of daily living (ADL), though outcomes vary considerably between users and device types.^2,3^ The global need for prosthetic services is growing due to factors such as aging populations, rising rates of diabetes-related amputations, and increasing instances of traumatic injuries.^4–6^ Ensuring that individuals have access to high-quality, well-fitting devices is essential for their well-being, yet traditional fabrication methods present several challenges related to resource requirements, cost, and accessibility.^1^

Prosthetic devices typically incorporate a custom interface, most commonly a socket, which transfers load between the residual limb and the prosthetic components. Conventional fabrication methods for these individualized components require skilled clinicians to manually craft each device through a series of steps including impression taking, cast rectification, socket fabrication, assembly, and fitting/alignment. This labor-intensive workflow is costly and time-consuming for both clinicians and patients, limiting accessibility across healthcare systems, particularly in rural and low-resource settings.^7–10^ Digital manufacturing methods have emerged as viable alternatives, including computer-aided design/computer-aided manufacturing (CAD/CAM) systems that use computer-controlled milling to carve device components, and additive manufacturing (also known as 3D printing), which builds parts layer by layer.^11–14^ Both approaches are typically paired with 3D scanning for patient anatomy capture. By enabling rapid fabrication and iterative adjustments based on digital patient data, these technologies offer the potential to improve device fit and patient comfort, while supporting greater customization, precision, and production efficiency.^11–16^

Additive manufacturing encompasses several distinct technologies that vary considerably in their printing mechanism, materials, cost, and output quality. Fused deposition modeling (FDM) extrudes heated filament through a nozzle and is generally the lowest-cost approach, though large variability in print quality exists.^17,18^ Stereolithography (SLA) uses an ultraviolet laser to cure liquid resin layer by layer, producing parts with high dimensional accuracy and smooth surface finishes at a relatively low cost.^17^ Selective laser sintering (SLS) and Multi Jet Fusion (MJF) both fuse polymer powder, using a laser and infrared light with liquid agents, respectively.^17–19^ SLS and MJF printers have significantly higher costs, but can produce parts without visible layers and with strength comparable to injection-molded components.^18,19^

Despite the growing body of research on digital manufacturing in prosthetics and orthotics (P&O), most studies emphasize technical aspects rather than clinical efficacy and impacts on patient clinical outcomes.^11,14,20^ As a result, there remains a lack of consolidated evidence regarding the real-world benefits and limitations of 3D-printed P&O devices from a clinical outcomes perspective. Addressing this gap is essential to inform clinical decision-making, manufacturing investments, and future research directions.

Several previous reviews have explored various aspects of digital manufacturing in prosthetics. In a review that included studies up to June 2018, Ribeiro et al.^11^ highlighted the limited research at the time, particularly with respect to real-world use. Savsani et al.^21^ provided an overview of additive manufacturing in prosthesis development, focusing on material science and mechanical properties. More recently, Oldfrey et al.^14,20^ performed a comprehensive review of digital fabrication techniques applied to P&O development. Their findings underscored significant knowledge gaps in the long-term effects of digital manufacturing on patient mobility, rehabilitation, and health outcomes, while also highlighting a rapid acceleration in research from 2016 onward.

Building on this prior work, the aim of the present scoping review was to focus specifically on clinical outcomes associated with additive manufacturing of prosthetic devices. This review differs from prior work in that it defined clinical outcomes as the central eligibility criterion for inclusion, rather than surveying fabrication techniques, material properties, or digital manufacturing more broadly. Given the anticipated heterogeneity in study designs, populations, and outcome measures within this emerging field, a scoping review methodology was selected in preference to a systematic review. This approach allowed the review to map the extent, range, and nature of the existing evidence and to identify key gaps, rather than to appraise study quality or synthesize pooled effect estimates, which would be premature given the current state of the literature.^22^ This review therefore sought to map clinical outcomes associated with 3D-printed prostheses, and to identify key gaps in the evidence base to inform both clinical practice and future research.

## METHODOLOGY

This scoping review was conducted in accordance with the JBI methodology for scoping reviews.^23^ An a priori protocol was registered with the Open Science Framework (OSF) (https://doi.org/10.17605/OSF.IO/3WXUR). Changes to the registered protocol were made during the conduct of the review; these are described below:

- The scope was refined to focus on additive manufacturing. While the authors’ original intentions were to focus on additive manufacturing (i.e., 3D printing technologies), the term “digital manufacturing” was used to have a broad scope. However, it became apparent during pilot screening that using “digital manufacturing” would be challenging, as it includes vastly different technologies and workflows, such as Computer Numerical Control (CNC) milling. This change in scope occurred following pilot screening, prior to full title/abstract screening.
- The original protocol outlined a broader scope that included both prosthetic and orthotic devices. However, following full text review and formal study selection, no studies with orthoses met the inclusion criteria, which required a limb loss or limb difference population. The scope was therefore narrowed at that time to include only prosthetic devices.
- The original protocol specified an inclusion date up to March 31, 2025. This was extended to December 31, 2025, as the initial data extraction was completed in early 2026. This allowed for additional recent studies to be included.

These deviations were not documented in the OSF registration at the time they were made, and the registered protocol was not subsequently amended or re-registered.

They are reported here in full so that the conduct of the review can be assessed against the registered protocol.

Reporting was conducted in accordance with the Preferred Reporting Items for Systematic Reviews and Meta-Analyses extension for Scoping Reviews (PRISMA-ScR)^24^ standards (**Figure 1**).

**Figure 1:**
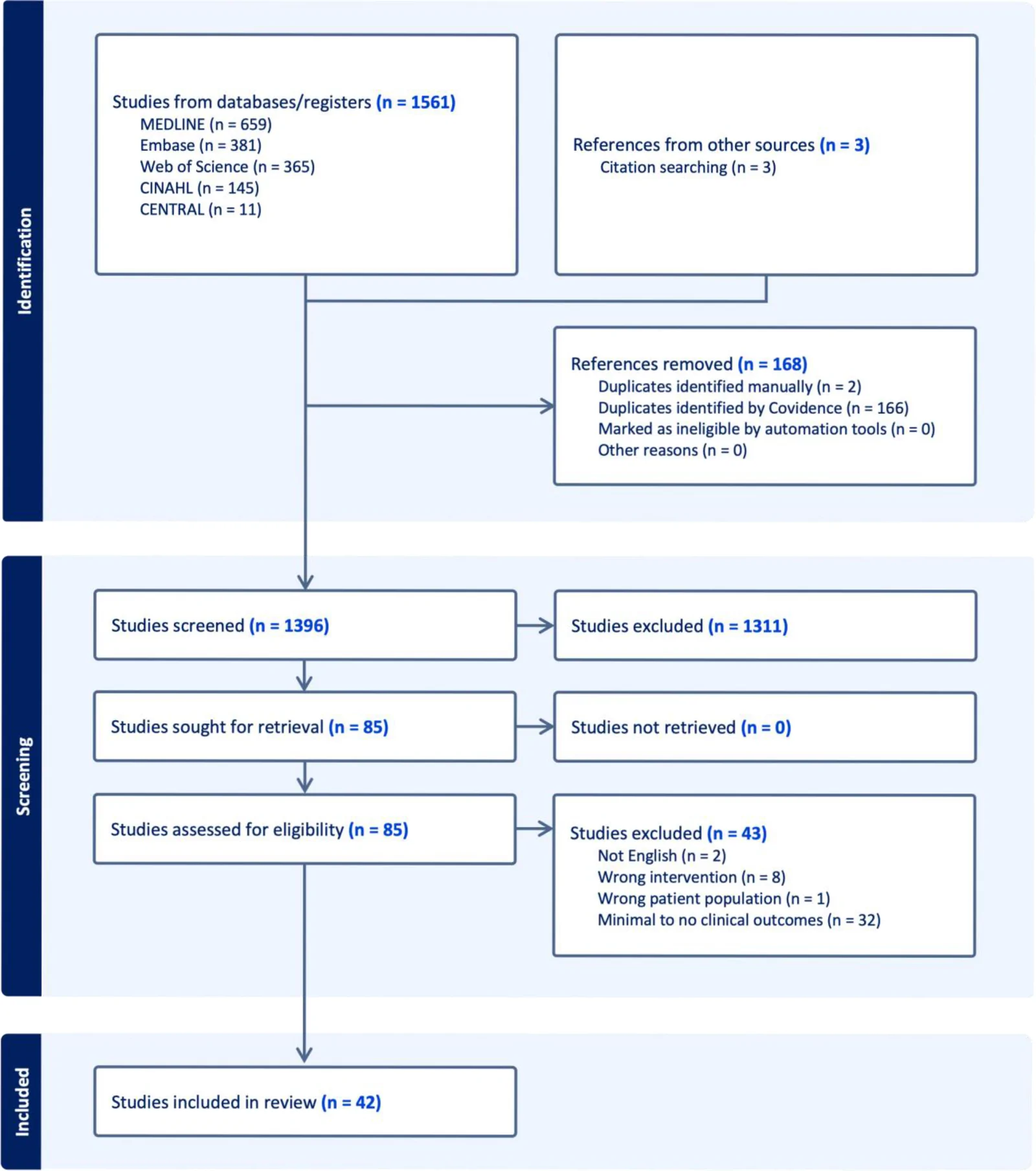
Preferred Reporting Items for Systematic Reviews and Meta-Analyses (PRISMA) diagram of search protocol.

### REVIEW QUESTION

What are the clinical outcomes associated with the use of additively manufactured (3D-printed) prostheses in individuals with limb loss or congenital limb differences?

### INCLUSION CRITERIA

#### Participants

Individuals of all ages with congenital limb differences or who have undergone limb amputation.

#### Concept

Clinical outcomes (e.g., functional performance, comfort, patient satisfaction, quality of life) with the use of a functional limb prosthesis directly created with additive manufacturing. Clinician- and patient-reported outcomes were included.

To be considered as reporting a clinical outcome, a study had to systematically assess the outcome using an identifiable method of measurement or elicitation, such as a named outcome measure or questionnaire, or a quantifiable functional or performance-based test (e.g., grip strength, range of motion, or gait parameters). Studies that described patient use of the device only in narrative or anecdotal terms, such as a general comment that the device was comfortable or easy to use, or a description of tasks or activities the patient performed without a defined protocol for assessing performance, were not considered to report a clinical outcome and were excluded. Similarly, outcomes that characterized the mechanical or biomechanical performance of the device itself, such as socket interface pressure, stress distribution, or material and structural testing, were considered technical rather than clinical outcomes, even when measured on a study participant, and were excluded unless reported using an established clinical measure such as a comfort or pain scale. Studies excluded for either reason were documented in the PRISMA-ScR flow diagram as having “minimal to no clinical outcomes.”

A prosthesis was considered functional if it was designed to restore or augment a specific physical capacity: upper limb prostheses were required to assist with prehension, grasp, or other purposeful hand and upper limb function, while lower limb prostheses were required to support ambulation or weight-bearing. Devices intended solely for aesthetic restoration of limb appearance were classified as cosmetic and excluded. For example, a silicone digit restoration intended solely to restore the appearance of the hand was excluded, whereas one designed to assist with object stabilization or prehension was considered functional and eligible.

Prostheses manufactured from 3D-printed molds were classified as a hybrid fabrication approach, since the final prosthetic device was not itself directly 3D-printed, and were therefore excluded.

#### Context

All geographic and clinical settings were included. Studies that did not evaluate clinical outcomes (e.g., those reporting only the mechanical or design characteristics of devices) or did not involve use of prostheses by individuals with limb loss or limb difference were excluded.

#### Types of Sources

The scoping review considered all study designs that include quantitative or qualitative clinical patient outcomes. This included but was not limited to experimental designs, observational studies, case-control studies and qualitative studies. Studies were required to be published in a peer-reviewed journal to be considered. Systematic reviews and other types of secondary research were not included.

### SEARCH STRATEGY

A three-step search strategy was used to identify relevant published studies. First, an initial limited search of MEDLINE (Ovid) was undertaken to identify relevant articles. Text words contained in the titles and abstracts of these articles, along with index terms used to describe them, were used to develop a comprehensive search strategy (**Supplementary Digital Content 1**) in consultation with an information specialist. This strategy was adapted for use in MEDLINE, Embase, CINAHL, CENTRAL, and Web of Science. Reference lists of included studies were also screened for additional eligible studies.

Studies published in English up to December 31, 2025, were included. The search strategy was peer reviewed by a second information specialist according to the Peer Review of Electronic Search Strategies (PRESS) 2015 guideline.^25^ Only English-language studies were included due to feasibility constraints. Searches were conducted on April 15, 2025 and January 16, 2026. A corrected re-run of the same time-limited search was additionally conducted on August 14, 2026, after identification of a minor typo.

#### Study Selection

Following the searches, all identified citations were collated, uploaded into Covidence (Veritas Health Innovation, Melbourne, Australia) and duplicates removed. A pilot test was conducted, with 30 titles and abstracts reviewed by two independent reviewers (CB and MH) and a third senior reviewer (AM) against the inclusion criteria for the review. During the pilot, disagreements were discussed as a group, and eligibility criteria were refined until all three reviewers were satisfied with the criteria. No formal agreement statistic was used. Following the pilot, the remaining titles and abstracts were screened by the same two independent reviewers, with disagreements resolved through discussion with the senior reviewer. Potentially relevant sources were retrieved in full.

Full-text articles were assessed independently by the same two reviewers for final inclusion. Reasons for exclusion at the full-text stage were recorded. Any disagreements at any stage of the selection process were resolved through discussion with the senior reviewer.

#### Data Extraction

Data were extracted independently by the same two reviewers using a data extraction tool adapted from the JBI Manual for Evidence Synthesis.^23^ Extracted data included details on participant characteristics, prosthetic type, additive manufacturing technique, study design, clinical outcomes assessed, and key findings relevant to the review question. The data extraction tool was piloted for 5 studies. Minor adjustments to the tool were made following the pilot extraction. Discrepancies were resolved through discussion with the senior reviewer.

#### Data Analysis and Presentation

Given the anticipated heterogeneity in study designs and outcome measures, data were synthesized descriptively. Critical appraisal of included sources was not conducted, consistent with JBI guidance for scoping reviews, as the aim was to map the extent and nature of the evidence rather than assess methodological quality.

## RESULTS

### STUDY SELECTION

The search yielded 1,396 studies after de-duplication. Following title/abstract screening, 85 potentially relevant full texts were retrieved. After full-text review against inclusion criteria, 42 studies were included in the review. Forty-three studies were excluded at full-text stage for: minimal to no clinical outcomes (n = 32; predominantly technical/engineering-focused studies describing device design, fabrication, or mechanical performance, with at most an incidental, non-systematically assessed comment on patient use or comfort), wrong concept/intervention (n = 8; examples include 3D-printed molds or non-3D-printed devices), non-English publication (n = 2), and wrong population (n = 1; not limb loss or limb difference). The PRISMA-ScR flow diagram is presented in **Figure 1**. Study-level characteristics and summarized findings of all included studies are presented in **Supplementary Digital Content 2**.

### STUDY CHARACTERISTICS

#### Study Methodology

The 42 included studies comprised 18 case reports,^26–43^ 13 case series,^44–56^ 4 pilot/feasibility studies,^57–60^ 1 longitudinal cohort study,^61^ 2 non-randomized experimental studies,^62,63^ 3 qualitative studies,^64–66^ and 1 cross-sectional study.^67^ No randomized controlled trials were identified. Except for two studies with n = 61 and n = 76,^62,67^ sample sizes were small, ranging from 1–11 (overall mean: n = 7.5; median: n = 4). **Figure 2** presents a timeline of included studies and **Table 1** presents the distribution by study design.

**Figure 2:**
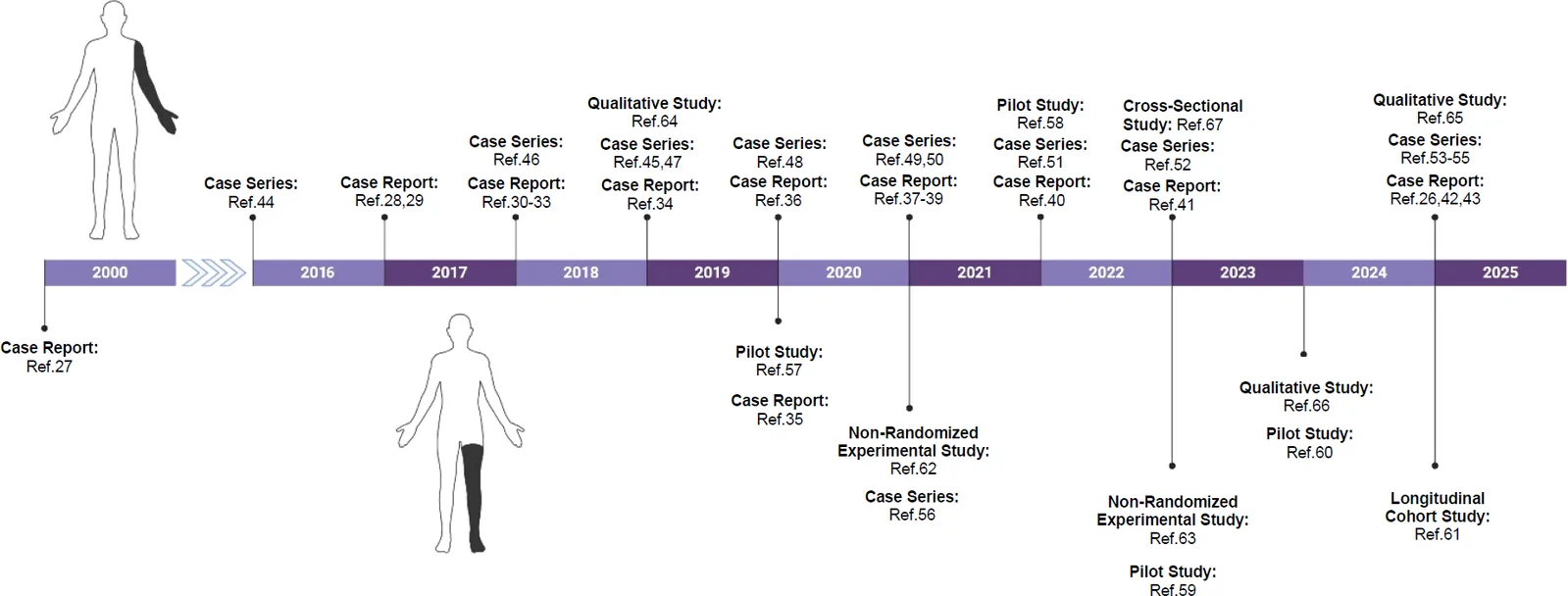
Timeline of included studies by publication year and study design.

**Table 1:** Study designs.

Study Design	TotalStudies	Upper Limb Studies	Lower Limb Studies	Average Participant Number (range)	Key Characteristics	Studies
Case Report	18	16	2	1	Single-patient device descriptions	26–43
Case Series	13	12	1	6 (2–11)	Small groups with heterogeneous outcomes	44–56
Pilot/Feasibility Study	4	1	3	9 (9–10)	Feasibility/acceptability focus; limited generalizability	57–60
Longitudinal Cohort Study	1	0	1	9	Longitudinal observation; no comparison group	61
Non-RandomizedExperimental	2	0	2	36 (10–61)	Comparative but non-randomized; short follow-up	62,63
Qualitative Study	3	2	1	8 (7–9)	Exploratory interviews; small sample	64–66
Cross-Sectional Study	1	1	0	76	Large single-timepoint sample; limited depth	67
Total	42	32	10			

#### Geographic and Temporal Distribution

The included studies were conducted across 20 countries, with the largest number originating from the United States (n = 13). Several studies were conducted in low- and middle-income countries by World Bank Classification,^68^ including Sierra Leone (n = 4) and India (n = 3). One study was conducted across multiple sites in Uganda, Tanzania, and Cambodia (**Figure 3**).^62^

**Figure 3:**
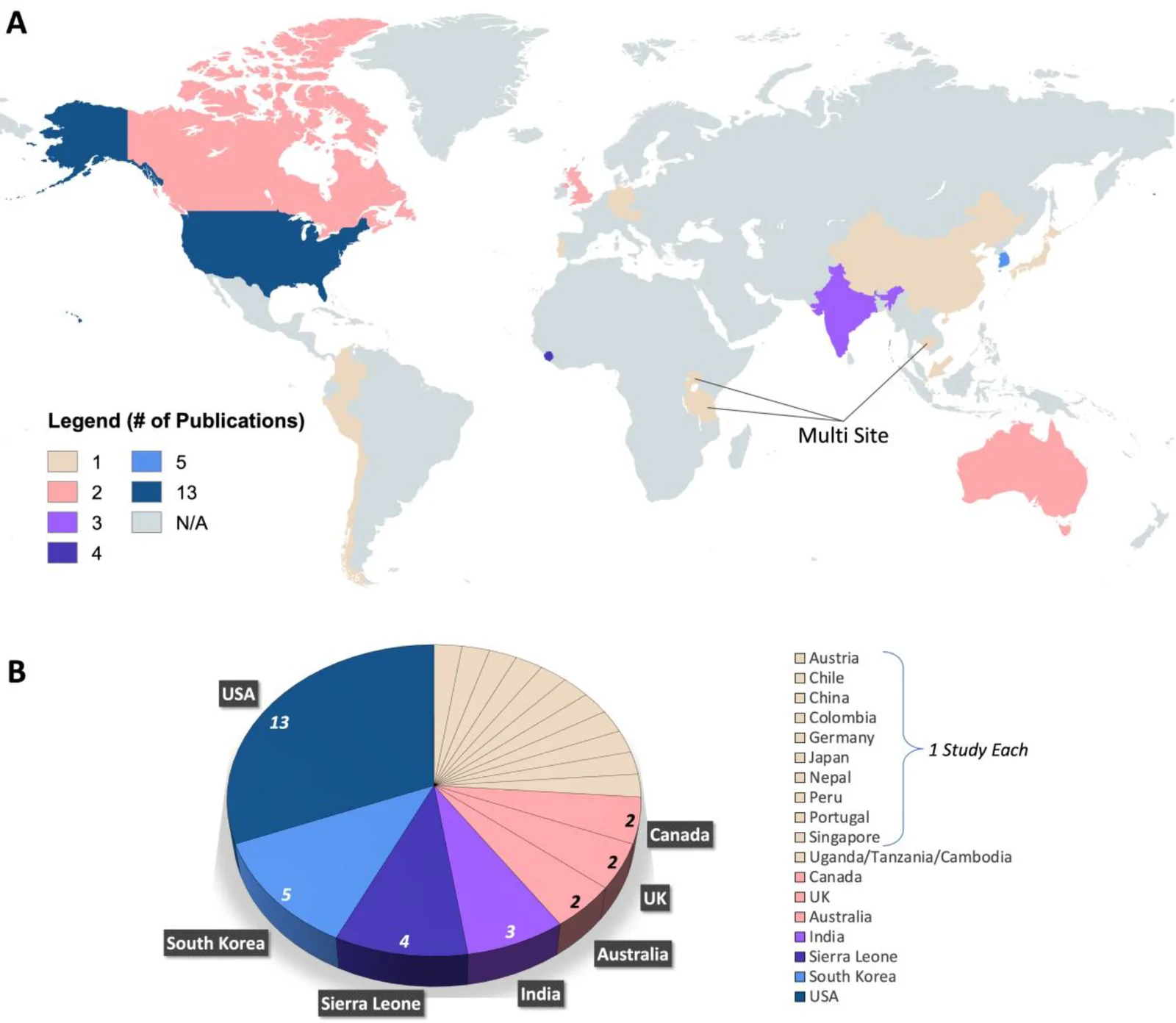
Geographic distribution of studies by (**A**) map view (created with mapchart.net under CC BY-SA 4.0 open license) and (**B**) pie-chart plot.

Publication year ranged from 2000 to 2025, with all but one from 2016 and later: 12/42 (28.6%) in 2016–2019, 14/42 (33.3%) in 2020–2022, and 15/42 (35.7%) in 2023–2025 (**Figure 4**).

**Figure 4:**
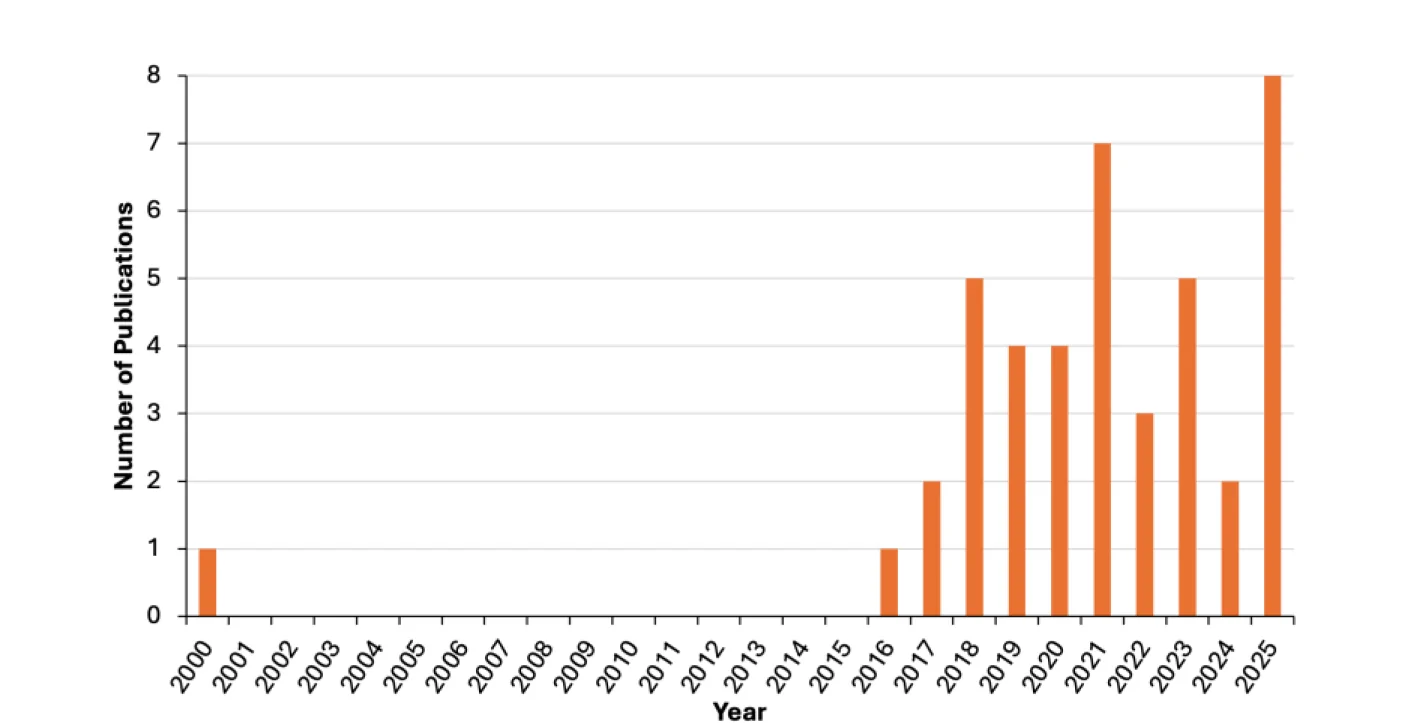
Number of included studies by publication year.

#### Anatomical Distribution and Demographics

The majority of studies (n = 32/42; 76.2%) examined prostheses for upper limb loss. This included 13 partial hand, 1 full hand, 10 transradial, 1 forequarter, and 7 studies with a mix of multiple classifications. There was a wide range of ages (both children and adults), genders (more male than female), and etiologies (primarily trauma and congenital).

All ten lower limb prosthetic studies examined transtibial amputation/limb loss. One study included children and young adults,^62^ with the others only including adults. Seven studies did not specify etiology of limb loss. Where specified, limb loss etiologies included trauma, vascular disease, infection, malignancy, and iatrogenic. Among these studies, the male to female ratio was approximately 1.7:1. **Table 2** summarizes the anatomic distributions and various demographic characteristics of the included studies.

**Table 2:** Anatomical distribution of studies.

Anatomical Focus	N Studies	Total Participants*	Age Range	Sex Distribution	Typical Etiology	Studies
Digit/Partial Hand	13	19	3–72	15 M, 4 F	Majority trauma, few congenital, other or undefined	30–34,36,37,39,42–44,46,51
Full Hand	1	1	8	1 M	Trauma	29
Transradial	10	45	4–64	33 M, 12 F	Balance of trauma and congenital, few infection and other etiologies	26,28,40,41,47,52–55,5
Forequarter	1	1	60	1 M	Trauma	38
Mixed Upper Limb	7	124	3–59	55 M, 58 F, 11 undefined	Balance of trauma and congenital, few infection and other etiologies	45,48–50,64,65,67
Transtibial	10	127	5–84	74 M, 43 F, 10 undefined	Balance of trauma, vascular, infection. Multiple studies did not specify	27,35,56,57,59–63,66
Total	42	317	3–84	179 M, 117 F, 21 undefined	Mixed	

* Some participants may be counted more than once, due to overlap of cohorts between studies.

### ADDITIVE MANUFACTURING METHODS

**Table 3** summarizes the additive manufacturing methods, including printing technology, printing material, and scanning methods. Detailed study-level data are provided in **Supplementary Digital Content 2**.

**Table 3:** Additive manufacturing techniques and materials.

Technique	N Studies	Upper LimbStudies	Lower LimbStudies	Primary Materials	Scanning Method	Studies
FDM	32	26	6	16 PLA, 6 ABS, 1 Polyamide, 8 Composite/Multiple materials/Other, 1 Undefined	11 Handheld, 6 Image-based, 1 3D Medical Images, 10 No scanning, 4 Undefined	26,28–34,36,37,39,40,42,44–46,48–56,59–62,64,66,67
SLS	3	1	2	2 Polyamide, 1 Composite/Multiple materials	1 Handheld, 1 Plaster mold digitization, 1 No scanning	27,43,63
MJF	1	1	0	Polyamide	Handheld	38
Undefined	6	4	2	5 Undefined, 1 Polyamide	3 Undefined, 2 No scanning, 1 Laser scanning custom-molded socket liner	35,41,47,57,58,65

#### Printing Technology

The majority of studies (n = 32/42; 76.2%) employed fused deposition modeling (FDM) for additive manufacturing. Selective laser sintering (SLS) was used in three studies. One study used Multi Jet Fusion (MJF). Six studies did not report the additive manufacturing method.

#### Printing Material

The most commonly used material was polylactic acid (PLA) (n = 16/42; 38.1%), though durability concerns were noted including heat-related deformation^26,44,45^ and moistureinduced brittleness.^45^ Other materials included acrylonitrile butadiene styrene (ABS) (n = 6/42; 14.3%), polyamide (n = 5/42; 11.9%), and multiple materials, composite or specialized filaments (n = 9/42; 21.4%). Six studies did not report the specific material(s) used.

#### Scanning Method

Handheld scanners were used for shape capture in 13 studies (n = 13/42; 31.0%), with workflows typically involving CAD software to create and refine a 3D model of the residual limb geometry. Six studies (n = 6/42; 14.3%) used image-based methods (2D photographs or photogrammetry), one study used 3D medical images (CT or MRI), and two studies scanned a traditional plaster mold or custom-molded socket liner. Thirteen studies did not employ any scanning/shape capture (n = 13/42; 31.0%), and seven studies did not define the scanning method.

### COMPARATORS

Fourteen studies (7 upper limb and 7 lower limb, n = 14/42; 33.3%) compared 3D-printed devices to conventionally manufactured prostheses worn by the same participant.^27,28,34,35,37,38,40,52,57–60,62,63^ One study also compared outcomes with an able-bodied control.^35^ In all other cases, participants only used the 3D-printed device.

### CLINICAL OUTCOMES

#### Upper Limb

Among upper limb studies (n = 32), there was large heterogeneity in both the outcome measures used and the study results. The type of device also varied greatly, ranging from small partial-finger prostheses to complex myoelectric prostheses.

Studies assessed participants’ objective performance and patient-reported function using various methods, outlined in **Table 4**. The most common objective performance measures were the Box and Block Test (n = 10/32, 31.3%), and Jebsen-Taylor Hand Function Test (JTHFT, n = 5/32, 15.6%).

**Table 4:** Standardized outcome measures reported.

Outcome Measure	Number of Studies	Studies
**Upper Limb**		
*Objective Performance Measures*		
Box and Block Test	10	28,30,31,34,37,40,45,46,50,51
Jebsen-Taylor Hand Function Test (JTHFT)	5	31,32,43,51,55
Grip Strength	3	28,37,43
Wrist Strength	2	44,45
Southampton Hand Assessment Protocol	2	52,67
University of New Brunswick Test of Prosthetic Function (UNB test)	2	29,55
Joint Range of Motion	3	41,44,51
Unilateral Below Elbow Test (UBET)	1	50
Brief Activity Measure for Upper Limb Amputees (BAM-ULA)	1	54
Nine-Hole Pegboard Test	1	28
Bimanual Coordination Tray Test	1	40
Bruininks-Oseretsky Test of Motor Proficiency (BOT-2)	1	41
Prosthesis Wear Time (from accelerometer data)	1	58
*Prosthesis/Limb Function – Patient Reported*		
Orthotics and Prosthetics Users’ Survey (OPUS)	8	31,34,40,42,43,47,58,64
Quebec User Evaluation of Satisfaction with Assistive Technology (QUEST) 2.0 Survey	7	31,34,38,40,46,53,64
Trinity Amputation and Prosthesis Experience Scales-Revised	2	43,58
ABILHAND	2	41,50
Consumer Prosthesis Use and Satisfaction Questionnaire	1	37
Michigan Hand Outcomes Questionnaire (MHQ)	1	43
Child Amputee Prosthetics Project (CAPP) Score	1	29
*Quality of Life/Other*		
Modified Barthel Index (MBI)	1	43
Patient-Specific Functional Scale (PSFS)	1	30
System Usability Scale	1	26
EQ-5D-5L Questionnaire	1	49
Canadian Occupational Performance Measure (COPM)	1	51
*No Standardized Outcome Measure Reported*	5	33,36,39,48,65
**Lower Limb**		
*Objective Performance Measures*		
Gait Parameters/Kinematics (motion capture)	3	27,35,63
Step Count and Wear Time (from accelerometer)	1	61
2-Minute Walk Test	1	59
Pedal Pressure (pressure sensors)	1	35
Custom Timed Pool Activities (ramp test, treading water, laps, etc.)	1	57
*Prosthesis/Limb Function – Patient Reported*		
Prosthesis Evaluation Questionnaire (PEQ)	2	59,62
Socket Comfort Score (SCS)	3	59,60,66
Orthotics and Prosthetics Users’ Survey (OPUS)	1	61
Prosthetic Limb Users Survey of Mobility	1	59
Amputee Body Image Scale-Revised (ABIS-R)	1	59
*Quality of Life/Other*		
EQ-5D-3L	1	61
Activities-Specific Balance Confidence Scale (ABC)	1	59
*No Standardized Outcome Measure Reported*	1	56

**Footnote:** Counts reflect studies using each named, standardized outcome measure; studies appear more than once where multiple measures were used. Many studies also reported study-specific or non-standardized outcomes, which are listed in Supplementary Digital Content 2 and are not catalogued here.

The most commonly used patient-reported outcome measure tools were the Orthotics and Prosthetics Users’ Survey (OPUS, n = 8/32, 25%) and the Quebec User Evaluation of Satisfaction with Assistive Technology 2.0 survey (QUEST 2.0, n = 7/32, 21.9%).

A small number of studies examined quality of life measures, such as the EQ-5D-5L questionnaire. Participants also frequently provided qualitative feedback using custom questionnaires or an unstructured format, particularly in the case reports and case series.

The large heterogeneity in patient populations, prosthetic devices, outcome measures, and results makes it challenging to report these clinical outcomes. However, several trends emerged. In studies without a comparator, participants frequently demonstrated improvements in gross motor function compared to using no prosthesis (e.g., increased Box and Block Test scores and successful completion of tasks such as carrying a laundry basket or hanging from a bar), while fine motor tasks often remained difficult.^39,50,54,55,67^ In several studies, mismatch between expectations and prosthesis capabilities contributed to reduced wear time or eventual device abandonment, particularly when precision tasks could not be performed.^50,65,67^ Overall, in these studies, the 3D-printed upper extremity prostheses were mostly viewed favorably, with generally improved clinical outcomes compared to baseline with no prosthesis, although a few case studies described mixed or even worsened scores on some patient-reported measures, including poor ability to perform lateral pinch-based tasks, pain, and decreased mood.^42,43,50^

In the seven studies where a traditionally manufactured prosthesis was used as a comparator, results were mixed rather than uniformly favoring one approach. Some reports found better fine motor control, pinch strength, or durability with conventional devices.^28,52,58^ Conversely, other studies showed higher satisfaction with 3D-printed devices, along with comparable or better dexterity on tests such as the Box and Block Test.^34,38^ Two studies had mixed results depending on the outcome, without a clear-cut preferred option.^37,40^

Across studies, 3D-printed devices were often rated positively for attributes such as weight and aesthetics, but concerns about grip strength, mechanical reliability, and long-term durability persisted.^34,36,40,45,51,65^

Several studies involved creating a custom 3D-printed prosthesis for a particular use case, such as playing an instrument or participating in sport. These included a guitarand cutlery-holding device for a child,^26^ a French horn support for a musician,^33^ and activity-specific devices for gymnastics.^39^ Across these reports, the 3D-printed devices enabled users to more easily complete previously challenging activities, although durability and independence in donning/doffing remained common limitations.

#### Lower Limb

While all ten lower limb studies examined participants with transtibial limb loss, there was again large heterogeneity in patient population, device type, and outcomes. Seven studies evaluated 3D-printed transtibial sockets used with standard prosthetic components distal to the socket.^27,56,59–62,66^ One study evaluated a cycling prosthesis,^35^ one study evaluated a 3D-printed prosthetic foot mounted on an otherwise conventional prosthesis,^63^ and another examined a 3D-printed swimming prosthesis used with a traditional socket.^57^

Studies assessed participants' objective performance and patient-reported function using various methods, outlined in **Table 4**. The most common objective performance measures were gait parameters and kinematics (n = 3/10, 30%). Other measures included the 2-minute walk test, measured step count and wear time from accelerometer data, cycling pedal pressure, and timed pool activities (all n = 1).

Patient-reported findings included the Prosthesis Evaluation Questionnaire (PEQ, n = 2), Socket Comfort Score (n = 3), Orthotics and Prosthetics Users’ Survey (n = 1), Prosthetic Limb Users Survey of Mobility (n = 1), Amputee Body Image Scale-Revised (ABIS-R, n = 1), EQ-5D-3L (n = 1), and Activities-Specific Balance Confidence Scale (ABC, n = 1).

Seven studies (n = 7/10, 70%) compared the 3D-printed device to a conventionally manufactured prosthesis. Most of the transtibial socket studies did not find statistically significant differences in gait kinematics, walking performance, and patient-reported outcomes between device types.^27,59,60^ Ratto et al. found that conventional transtibial prostheses were rated significantly higher than FDM printed sockets for ambulation and utility on the PEQ.^62^ Trinler et al.^63^ found that the 3D-printed prosthetic foot showed reduced ankle range of motion, lower plantar flexion power, and higher stiffness compared to conventional energy storing and returning feet. The two activity-specific devices performed well for their intended purposes: the cycling prosthesis improved kinematic alignment and reduced unwanted pedal pressure compared to a walking prosthesis,^35^ and the amphibious prosthesis reduced pool entry and exit time with comparable swimming lap times when compared to a standard swim ankle prosthesis.^57^

The three studies (n = 3/10, 30%) without a comparator,^56,61,66^ reported on device feasibility, use, and satisfaction rather than comparative performance. In two studies by van der Stelt et al., participants demonstrated high device adoption and wear time, with most achieving their rehabilitation goals.^56,61^ Patient-reported quality of life and functional status showed improvement trends over time, though these did not reach the authors’ predefined statistical significance threshold of p < 0.01.^61^ In the longer-term follow-up study, durability concerns were identified, with six of the nine FDM-produced PLA sockets sustaining damage over the 2.5-year follow-up period, requiring replacement of the sockets with a reinforced design that was not fully 3D-printed.^61^ In a primarily qualitative study, six of nine participants accepted their 3D-printed socket, and had high Socket Comfort Scores, while three rejected the socket and had comparatively lower comfort scores.^66^

## DISCUSSION

This scoping review mapped the current evidence on clinical outcomes associated with 3D-printed prosthetic devices in individuals with limb loss or congenital limb differences. Overall, the literature suggests that additive manufacturing is increasingly considered for prosthetic fabrication, particularly where customization, low-volume production, and activity-specific design are prioritized. At the same time, the evidence base remains clinically immature. While there are numerous technical and engineering reports of 3D-printed prostheses prior to 2016, these were excluded from our review due to the lack of structured clinical outcome reporting.^11,14,21^ There has been a rapid increase in studies that report outcomes since 2016 (**Figure 4**); most included studies were case reports, case series, or feasibility studies, and no randomized controlled trials (RCT) were identified (**Table 1**). While formal critical appraisal was not undertaken, this distribution of study designs suggests that technical innovation in this field continues to outpace rigorous clinical evaluation.

### CLINICAL FINDINGS AND IMPLICATIONS

#### Mixed Findings

A central finding of this review is that the available literature provides preliminary evidence of the feasibility and acceptability of 3D-printed prostheses, rather than clear clinical superiority over conventionally fabricated devices. Across both upper- and lower-limb applications, 3D-printed devices were often reported to be functional, acceptable, and associated with favorable user experiences. However, comparative findings were generally mixed, especially for lower-extremity prostheses, with many studies reporting similar outcomes between device types rather than consistent advantages for one fabrication approach.^27,37,40,59,60^ From a clinical end-user perspective, and with the current evidence lacking RCTs, additive manufacturing should not yet be assumed to directly improve outcomes simply because it offers digital customization or novel design freedom. Rather, current evidence suggests that it may represent a viable alternative prosthetic fabrication pathway in selected contexts.

The upper-limb literature was particularly diverse (**Table 2**), encompassing finger and partial-hand prostheses, full-hand devices, transradial devices, a forequarter prosthesis, and a range of task-specific designs. This diversity reflects a widely cited potential advantage of additive manufacturing: the ability of clinicians to create highly individualized devices with geometries and functions that may be difficult, time-consuming, or costly to achieve using conventional methods. However, cost, fabrication time, and manufacturing efficiency were not evaluated as outcomes in the included studies, and these remain theoretical advantages rather than findings of this review. The diversity of device types also contributed to substantial heterogeneity in study populations, device complexity, intended use, comparator type, and outcome assessment, limiting direct cross-study comparison (**Table 4**). As a result, while upper-limb prostheses may be one of the most promising areas for additive manufacturing, the current literature does not yet allow strong conclusions regarding which specific upper-limb indications benefit most.

Across the upper-limb studies, a recurrent pattern was that gross motor function and basic task performance were more frequently improved than fine motor control. Several studies described gains in measures such as the Box and Block Test or ADL completion, whereas precision-based activities often remained difficult.^50,54,55,67^ This suggests that many currently reported 3D-printed upper-limb devices may be better suited to supporting grasp, stabilization, or participation in broader functional activities than to restoring refined dexterity. Some studies reported reduced wear time or abandonment when user expectations exceeded device capability, particularly for precision tasks.^50,65,67^ These findings underscore the importance of careful patient selection, realistic goal setting, and transparent communication regarding the likely functional role of a prosthesis.

Notably, patient satisfaction did not always align directly with objective performance. Even when functional comparisons with conventional devices were mixed, 3D-printed prostheses were often rated positively for attributes such as light weight, appearance, and personalization.^34,36,40,45,51,65^ This reinforces the importance of patient-centered evaluation in prosthetics research.

By contrast, the lower-limb literature, which was focused entirely on transtibial prostheses (**Table 2**), suggested that comparable rather than superior outcomes may be the more realistic near-term benchmark. Several comparative studies found similar gait and patient-reported outcomes between 3D-printed and conventionally manufactured sockets, with minimal differences across many measures, though none were designed as formal equivalence or non-inferiority trials.^27,59,60^ This is still clinically relevant; if a 3D-printed socket can achieve comparable mobility and comfort outcomes, then the potential advantages of additive manufacturing may lie less in superior biomechanics and more in service delivery, including streamlined fabrication, reproducibility, and potential improvements in access.^11,12,69,70^ However, these service delivery advantages were not evaluated in the included studies, and some expert opinion cautions that anticipated cost savings and workflow efficiencies are often overestimated.^71^ Furthermore, not all comparative findings were neutral: one study reported significantly higher ratings for ambulation and utility with conventional prostheses,^62^ and another found less favorable biomechanical performance in a 3D-printed prosthetic foot compared with a conventional design.^63^ Taken together, these findings suggest that lower-limb applications may be feasible, but performance likely depends heavily on device design, material choice, clinical setting, and the demands of the intended use.

#### Activity-Specific vs General-Purpose

A recurring theme in this review was the use of activity-specific prostheses. In the upper limb, examples included devices designed for playing guitar, holding cutlery, playing a French horn, and participating in gymnastics.^26,33,39^ In the lower limb, task-specific devices such as cycling and water prostheses also performed well for their intended purposes.^35,57^ While these reports are largely proof-of-concept case reports and should not be interpreted as evidence of effectiveness, they demonstrated potential utility in addressing individualized, niche, or low-volume needs that are not well served by standard commercial components or traditional workflows.

#### Technical Aspects

Durability and mechanical reliability emerged as recurring concerns across both upper- and lower-limb studies. These included grip strength, structural robustness, long-term wear, and device damage over time.^34,36,40,44,45,51,56,61,65^ Such concerns are particularly important in prosthetics, where repeated loading, environmental exposure, and daily use impose substantial demands on devices. The longer-term follow-up study of 3D-printed transtibial sockets is especially noteworthy, as it documents socket damage in several cases.^61^ This suggests that short-term feasibility or early patient satisfaction should not be interpreted as sufficient evidence of long-term clinical readiness. Greater attention to longitudinal follow-up, failure modes, repair burden, and maintenance requirements will be essential for future work.

The predominance of FDM and low-cost materials such as PLA and ABS likely reflects the accessibility and affordability of these manufacturing approaches (**Table 3**), but may also contribute to some of the limitations observed clinically.^26,45^ Low-cost workflows may facilitate innovation and expand access, yet they may not always provide the consistency, durability, or mechanical performance required for prolonged prosthetic use. At the same time, technical reporting across studies was often incomplete, with some articles failing to specify printing method, material, or scanning workflow. This limits reproducibility and makes it difficult to determine whether variable outcomes reflect differences in digital rectification, shape capture, or manufacturing quality. More detailed and standardized reporting of fabrication parameters would strengthen both research synthesis and clinical translation.

### METHODOLOGICAL GAPS

#### Lack of Standardized Outcome Measures

This review also highlights an important methodological challenge in the field: substantial heterogeneity in outcome measure selection and reporting. Studies used a wide range of objective performance measures, patient-reported outcome measures, qualitative feedback methods, and custom questionnaires (**Table 4**). While this reflects the diversity of prosthetic devices and patient goals, it limits evidence synthesis and comparison across studies.

The broader prosthetics field has made concerted efforts to address this through consensus-based frameworks.^72,73^ For lower limb prosthetics, the COMPASS initiative (2023) proposes a standardized battery including the Amputee Mobility Predictor (AMP), Timed Up and Go (TUG), and Two Minute Walk Test (2MWT) as performance measures, alongside the Prosthesis Evaluation Questionnaire (PEQ) and Trinity Amputation and Prosthesis Experience Scales-Revised (TAPES-R) as patient-reported measures, with further measures recommended depending on functional level and rehabilitation context.^72^ For upper limb prosthetics, the ULPOM working group (2009) developed an ICForganized framework identifying a toolkit of validated measures across function, activity, and participation domains.^73^ For functional assessment, only the Assessment of Capacity for Myoelectric Control was formally recommended, with several additional tools including the Box and Block Test and Jebsen Hand Function Test categorized as warranting further validation to be acceptable for use in upper limb prosthetics.^73^

This review found limited alignment with these frameworks. Among lower-limb studies, the AMP and TUG did not appear in any study, while the 2MWT and PEQ were each used in only one and two studies, respectively. However, it should be noted that most studies predate the publication of the 2023 COMPASS recommendations. In upper-limb studies, all of which postdate the ULPOM framework, no single measure was used in a majority of studies. The Box and Block Test was the most common performance measure, appearing in one-third of studies, while the OPUS and QUEST 2.0 were the most common patient-reported measures, each appearing in approximately one-quarter of studies. However, all three fall only within the ULPOM “consider” categorization, and many studies relied on custom questionnaires, further limiting comparability.

Future research would benefit from adoption of a core set of standardized outcome measures aligned with established frameworks, supplemented with additional device- and goal-specific measures. Of particular relevance, COMPASS acknowledges that outcome measures suited to low- and middle-income country contexts remain underdeveloped, a gap that is especially pertinent given additive manufacturing's potential role in expanding prosthetic access in these settings.^72^ Beyond these frameworks, standardized reporting of device-specific outcomes, including comfort, satisfaction, mechanical durability, device abandonment, and adverse events, would more completely characterize outcomes with 3D-printed prostheses. Doing so would also facilitate comparisons across device classes and clinical settings, strengthening the evidence base available to future systematic reviews and meta-analyses.

#### Longitudinal Evidence Gaps

Long-term, real-world evidence remains one of the most important gaps in the prosthetic additive manufacturing literature. Most studies involved small samples and short follow-up periods, meaning there is still limited evidence regarding sustained use, abandonment, component survival, repair frequency, and the impact of 3D-printed prostheses on rehabilitation trajectories over time.^14,21,56,61^ This is particularly relevant given that some of the perceived advantages of additive manufacturing, such as rapid iteration and ease of replacement, may only become meaningful when evaluated longitudinally within the actual device lifespan. Accordingly, the field would benefit from larger prospective and comparative studies designed to assess clinical outcomes over meaningful follow-up periods. Where randomization is feasible, RCTs would strengthen the evidence base. However, given the individualized nature of many prosthetic applications, alternative designs including pragmatic trials, N-of-1 studies, standardized registries, and longitudinal cohort studies may be equally or more appropriate in many contexts.

### STRENGTHS, LIMITATIONS, AND FUTURE DIRECTIONS

The present review has several strengths. It focused specifically on clinical outcomes, which addressed an important gap in prior reviews that emphasized technical development and manufacturing considerations. The review followed JBI methodology, used a PRESS-reviewed search strategy, and incorporated independent screening and data extraction.

Several limitations should also be acknowledged. First, only peer-reviewed studies were included, meaning grey literature sources, including conference proceedings, theses, and technical reports were excluded, which is a departure from JBI guidance. While relevant evidence may exist outside the peer-reviewed literature, grey literature sources typically provide limited methodological detail and were considered unlikely to yield a substantial number of additional eligible studies given the requirement for a clinical outcome measurement or elicitation. Second, only English-language studies were included, which may introduce language bias by excluding potentially relevant studies published in other languages. Third, consistent with scoping review methodology, critical appraisal of included studies was not performed, so the review mapped the extent and nature of the evidence without assessing its methodological quality. Readers should interpret the findings with this in mind: the predominance of case reports and case series among included studies means the evidence base is subject to substantial risk of bias, especially given that case reports describing successful or novel 3D-printed devices are likely more frequently published than reports of unsuccessful attempts or unfavorable outcomes. Furthermore, without formal methodological appraisal, findings from single case reports and stronger comparative studies contribute similarly to the narrative synthesis, meaning the apparent consistency or direction of findings should not be interpreted as certainty of evidence. Results were synthesized descriptively to map the nature and extent of reported outcomes rather than to draw conclusions about treatment efficacy or comparative effectiveness. Overall, the present review should be considered a stepping stone until a larger body of clinical evidence becomes available to support more rigorous systematic reviews and meta-analyses.

The growing demand for P&O services, combined with the labor intensity, cost, and access barriers associated with conventional fabrication, has motivated interest in digital workflows.^1,4–6^ If 3D-printed prostheses can achieve comparable outcomes in selected contexts, this may support their potential role in improving access and enabling tailored solutions where conventional fabrication is difficult to deliver efficiently. This may be particularly relevant in low-resource, time-constrained, or geographically dispersed settings, where several of the included studies were conducted (**Figure 3**). Nevertheless, enthusiasm should be balanced by the need for adequate durability, user follow-up, and clinical oversight.

## CONCLUSION

Additive manufacturing is an emerging and clinically promising approach for prosthetic fabrication. This scoping review found that the current evidence base is growing, but remains dominated by small, early-stage studies with substantial heterogeneity in device type, study design, and outcome assessment. Across the available literature, 3D-printed prostheses were fabricated, fitted, and used across a range of clinical contexts, and were often associated with favorable user experiences, improvements in gross motor function when compared to no prosthesis, and successful participation in activities of daily living. However, findings were mixed when compared with those of conventionally manufactured prostheses, and concerns related to fine motor performance, durability, and long-term reliability remain. Overall, the current evidence provides preliminary support for 3D-printed prostheses as a fabrication strategy for highly individualized or task-specific devices, as well as in care settings where conventional fabrication is limited by cost, time, or access barriers, though whether these advantages translate meaningfully in practice remains to be established. Given the rapid pace of development in 3D-printed prosthetics, the present review provides a timely synthesis of the available clinical evidence and serves as a foundation for future work. Larger comparative studies, longer-term follow-up, improved reporting of fabrication methods, and more standardized outcome measures are needed to better define the clinical impact of additive manufacturing in prosthetic care.

## Figures and Tables

**Table:** 

Study (Year, Primary Author, Country, Study Design)	Study Objective	N, Age Range, Gender, Anatomic Focus, Etiology	Digital Manufacturing Method, Materials, Scanning, and Comparator	Outcome Measures Used	Key Clinical Findings
2000, Rogers,^27^ USA, Case report	Test feasibility of using SLS printing technique to create a transtibial prosthetic socket	N = 1, Age 65, Male, Transtibial, Undefined etiology	Selective Laser Sintering, Polyamide, Plaster mold digitization, Compared to conventional device	Gait parameters/kinematics, VAS for comfort and ambulation	The SLS prosthesis demonstrated improved gait outcomes with 3% increased speed and better steplength symmetry compared to conventional socket. Socket comfort favored the SLS device (78 vs 68), though ambulation ease was comparable. No statistical analyses performed; concerns raised about long-term durability and structural integrity.
2016, Zuniga,^44^ USA, Case series	Identify anthropometric, active ROM, and strength changes after 6 months of using a wrist-driven 3D-printed transitional prosthetic hand for children with upper-limb deficiencies	N = 5, Age 310, 3 Male, 2 Female, Digit/partial hand, Trauma and congenital	FDM, PLA and ABS, No scanning, No comparator	Anthropometric measures, Active ROM, Wrist strength, Device usage survey	After 6 months of 3D-printed prosthesis use, there were significant increases in forearm circumference and wrist flexion/extension ROM. There was no statistically significant difference in wrist strength. 3/5 devices broke or malfunctioned.
2017, Lee,^28^ South Korea, Case report	Compare hand functions of a preexisting custom myoelectric prosthesis to a 3D-printed pressure-sensored prosthesis for an upper-extremity amputee	N = 1, Age 52, Male, Transradial, Trauma	FDM, PLA, Handheld scanner, Compared to conventional device	Box and Block Test, Grip strength, Nine-Hole Pegboard Test, Self-reported ADL performance	The 3D-printed prosthesis showed comparable dexterity performance to the myoelectric device but significantly lower grip strength (13 vs 30 lb). The patient completed all 5 ADLs with the myoelectric prosthesis but only 2/5 with the 3D-printed version. Patient reported dissatisfaction with the 3D-printed device due to poor aesthetics, short battery life, motor noise, and low durability when exposed to water or impacts.
2017, Xu,^29^ China, Case report	Describe a clinical case with detailed instruction of employing a novel 3D-printed prosthesis in a child after traumatic wrist amputation; Evaluate short-term function outcomes following periprosthetic rehabilitation; present comprehensive clinical guide for 3D-printed prosthesis	N = 1, Age 8, Male, Full hand/wrist, Trauma	FDM, ABS, Undefined scanning, No comparator	UNB Test of Prosthetic Function, Child Amputee Prosthetics Project (CAPP) Score	Following 6 months of healing and 3 months of structured rehabilitation, the child demonstrated significant functional improvements (UNB spontaneity and skill scores nearly tripled, p < 0.001). The child performed satisfactorily in various ADLs including eating, writing, self-dressing, and riding a bicycle. Parents reported high satisfaction with both device performance and ADL outcomes. The prosthesis remained awkward for fine and bimanual movements such as tying shoelaces despite training.
2018, Alvial,^32^ Chile, Case report	Quantitatively assess the impact of using 3D-printed siliconeembedded prostheses that are created within the clinical environment	N = 1, Age 51, Male, Digit/partial hand (amputation of all left-hand fingers at different levels), Trauma	FDM, PLA (internal structure embedded in silicone), 3D medical imaging (CT/MRI), No comparator	Jebsen-Taylor Hand Function Test, Qualitative work-related task performance	The prosthesis enabled improved performance in multiple JTHFT tasks including card turning, lifting small objects, simulated feeding, and manipulating large/light objects. The patient successfully performed work tasks previously impossible without prosthesis, such as driving nails and opening plastic bags, with no pain or skin irritation. Limitations included soft silicone being easily damaged by sharp objects, becoming dirty, and potentially dislodging when lifting heavy objects.
2018, Day,^33^ UK, Case report	Design, manufacture, and test a 3D-printed assistive device for a patient with a partial hand amputation to play the French horn	N = 1, Age not specified, Male, Digit/partial hand (left 5^th^ digit), Surgical complication post Dupuytren’s contracture release	FDM, PLA, Photogrammetry, Compared to prototype device	Pugh's Concept Matrix for patient satisfaction	Two of three printed devices demonstrated improved satisfaction over the baseline prototype. The patient reported being “very satisfied” with the final device iteration (weighted score 37), successfully enabling French horn playing.
2018, Lee,^31^ South Korea, Case report	Report a case of a patient with an amputated thumb, who wore specifically shaped finger prostheses that were optimal to perform specific movements and were produced by a 3D scanner and printer and allowed improved functioning	N = 1, Age 67, Male, Digit/partial hand (right thumb amputation above the metacarpophalangeal joint), Trauma	FDM, Flexible thermoplastic elastomer, Handheld scanner, No comparator	Jebsen-Taylor Hand Function Test (JTHFT), Box and Block Test, OPUS, QUEST 2.0	The patient demonstrated functional improvements across most measures, with BBT increasing from 17 to 21 blocks and 6 of 7 JTHFT tasks showing improvement (only stacking checkers declined). Patient reported high satisfaction (QUEST 36/40, mostly “very/quite satisfied”) and found most activities easy to perform (OPUS 16/19 items “easy/very easy”). Tying shoes and using scissors remained difficult.
2018, Swartz,^30^ USA, Case report	Develop custom voluntary-closing, body-powered thumb mechanism for partial-hand amputee	N = 1, Age 50, Male, Digit/partial hand (amputations of the index finger and thumb on the left), Trauma	FDM, ABS, No scanning, No comparator	Box and Block Test, Patient-Specific Functional Scale	The prosthesis enabled the patient to perform tasks previously impossible, progressing from 0 to 23 blocks on the BBT. All patient-identified functional goals (gardening with shovel, holding items during cooking, wringing mop) improved from “unable to perform” to “moderate difficulty” after three therapy sessions and device modifications. The device successfully enabled use of fishing rod, shovel, rake, and wheelbarrow with additional support mechanisms.
2018, Zuniga,^46^ USA, Case series	Describe the development of 3D-printed prostheses using antibacterial filaments and verify the antibacterial properties of the 3D-printed prostheses	N = 2, Age 40–65, Male, Digit/partial hand, Trauma	FDM, PLACTIVE (antibacterial PLA), 2D photographs, No comparator	Box and Block Test, QUEST 2.0	Both participants demonstrated significant functional improvements in dexterity, with BBT scores increasing by 26% and 54% respectively. Participants reported high satisfaction with the antibacterial prosthesis (QUEST scores >4.5/5, “quite to very satisfied”). The antibacterial properties were successfully verified through laboratory testing.
2019, Ku,^47^ South Korea, Case series	Evaluate the impact of low-cost 3D-printed myoelectric-interfaced prosthesis on transradial amputees' daily lives	N = 10, Age 22–63, 8 Male, 2 Female, Transradial, trauma/infecti on	Undefined 3D printing method, Undefined materials, Undefined scanning, No comparator	OPUS, VAS for Pain	After 3 months of prosthesis use, participants showed significant functional improvement with OPUS-UEFS scores increasing by approximately 14.6 points (45.50 to 60.10) and reduced pain levels (VAS 5.2 to 4.0). Patients with longer residual forearms demonstrated better functional outcomes, suggesting anatomical factors influence prosthesis effectiveness.
2019, Young,^34^ USA, Case report	Provide a detailed description of patient-specific body-powered 3D-printed partial finger prosthesis; compare to commercial prosthesis	N = 1, Age 72, Male, Digit/partial hand (amputation of the left index finger at the PIP joint), Trauma	FDM, PLACTIVE (antibacterial PLA), 2D photographs, Compared to conventional device	Box and Block Test, OPUS, QUEST 2.0	Both the 3D-printed and conventional prostheses demonstrated identical functional performance (BBT 18 to 22 blocks/min). The 3D-printed device achieved higher satisfaction ratings (QUEST 3.3 ± 1.2 vs 2.5 ± 0.5) and offered the advantage of not wearing or tearing clothing, though it was reported as less durable. Both devices enabled easy performance of basic ADLs like carrying laundry and donning/doffing.
2019, Zuniga,^45^ USA, Case series	Identify functional and strength changes after using 3D-printed transitional prostheses in children	N = 11, Age 3–15, 6 Male, 5 Female, Digit/partial hand and transradial, Trauma/congenital	FDM, PLA and ABS, Handheld scanner, No comparator	Box and Block Test, Wrist flexion/extension strength	Children demonstrated significant functional improvements with BBT scores more than doubling (6.27 to 13.00 blocks/min). Wrist strength increased but did not reach statistical significance. Most children (10/11) used the prosthesis for approximately 2 hours daily across multiple contexts including fun activities, home tasks, school, play, and sports.
2019, Zuniga,^64^ USA, Qualitative study	Describe a remote fitting procedure for upper limb 3D-printed prostheses and assess patient satisfaction and comfort with 3D-printed prostheses fitted remotely	N = 8 (6 Children, 2 Adults), Age 6–59, 5 Male, 3 Female, Digit/partial hand and transradial, Congenital/undefined	FDM, PLACTIVE (antibacterial PLA), 2D images, No comparator	OPUS, QUEST 2.0, Custom satisfaction survey	Participants reported being “quite satisfied” overall with remotely fitted prostheses, with highest ratings for weight, safety, dimensions, and ease of use (QUEST). Participants found the prosthesis weight manageable, aesthetically pleasing, easy to don, and well-fitting. The easiest tasks were donning/doffing and drinking from a cup, while dialing a touch-tone phone proved most difficult. Average comfort was moderate (2.25/5), and results on quality of life improvement were mixed.
2020, Alturkistani, ^36^ India, Case report	Design and develop affordable patient-specific partial hand prosthesis for developing countries using 3D-printing techniques	N = 1, Age not reported, Male, Digit/partial hand (transmetacarpal with middle ring little finger and distal palm missing), Trauma	FDM, PLA/TPU/Ninjaflex, Undefined scanning, No comparator	Grasp taxonomy test, Grasp and lift test	The patient successfully maintained grasp on 12 of 14 test items (unable to grasp CD and scissors) with maximum lift capacity of 700 g sustained for 3 seconds. The patient reported key advantages including compactness, light weight, and simple donning/doffing, expressing preference for this device over cosmetic prostheses and no-prosthesis options. Grip was achieved by using the contralateral hand to mold finger positions.
2020, Goldstein,^57^ USA, Pilot study	Measure ease of use and likability of novel 3D-printed amphibious lower limb prosthesis in pool; determine differences in time to complete basic swim tasks using the novel prosthesis or a standard Swim Ankle prosthesis	N = 10, Age 29–82, 8 Male, 2 Female, Transtibial, Undefined etiology	Undefined method, Polyamide, Undefined scanning, Compared to conventional device	Pool task performance, survey	The 3D-printed amphibious prosthesis significantly reduced time to enter and exit the pool compared to conventional devices, with comparable swimming lap times. The majority preferred the 3D-printed prosthesis for multiple attributes including weight, comfort, energy efficiency, balance, swimming quality, overall satisfaction, and reduced discomfort during aquatic activities.
2020, Tiele,^35^ UK, Case report	Develop a prototype prosthesis using a flexible ankle joint design for patients with transtibial lower-limb deficiency to use in amateur sports cycling	N = 1, Age not specified, Gender not specified, Transtibial, Undefined etiology	Undefined method, Undefined materials, Laser scanning custom-molded socket liner, Compared to conventional device and able-bodied control	Cycling kinematics, Pedal pressure	The cycling-specific prototype demonstrated substantial improvements over the walking prosthesis, including 64% reduction in bilateral ankle and heel kinematic misalignment and decreased unwanted pedal pressure during recovery phase (60N to 50N). The device increased maximum ankle range of motion from 7° to 18°, though this remained well below the healthy control average of 35°.
2020, van der Stelt,^48^ Sierra Leone, Case series	Investigate feasibility of using 3D printing in a resource-limited healthcare setting to fabricate customized prostheses, braces, and splints for patients with amputation, congenital abnormalities, or burns; assess patient satisfaction and functionality	N = 4, Age not specified, Gender not specified, Full hand/wrist, Transradial, Shoulder disarticulation, Trauma	FDM, PLA, Handheld scanner, No comparator	Satisfaction and function questionnaire, Hours of use	Participants demonstrated high device adoption with three patients using prostheses for more than 6 hours daily and one for 4 hours. All participants valued aesthetics equally with functionality and reported increased self-confidence and societal participation. The prostheses allowed participants to expose and use their hands in functional capacities in public settings, whereas they previously concealed their hands due to stigma.
2021, Anderson,^39^ USA, Case report	Investigate application of 3D-printed hand to improve child's participation, confidence, satisfaction in gymnastics	N = 1, Age 9, Female, Digit/partial hand, Congenital	FDM, PLA, No scanning, No comparator	Hang time, Task completion, 5-point Likert survey	The child demonstrated functional improvement with bar hang time increasing from 3 to 28 seconds, successfully completing chin-hold for 5 seconds and pull-over with minimal assistance. Participation, confidence, and satisfaction all improved from 2/5 to 4/5.
2021, Bhat,^50^ India, Case series	Evaluate functional outcomes of custom designed 3D-printed prosthesis in children with congenital upper limb differences	N = 11 (originally 14, 3 withdrew), Age 3–11, 5 Male, 6 Female Digit/partial hand and transradial, Congenital	FDM, PLA, No scanning, No comparator	UBET, Box and Block Test, ABILHAND	While BBT scores showed modest improvement (24 to 35, p = 0.049), both UBET and ABILHAND showed a decrease in score with the use of a prosthesis (20.4 to 14.7 for UBET, 25 to 20.7 for ABILHAND), explained by the lack of lateral and tripod pinch in the design of the prosthesis. Three participants withdrew from the study: two due to poor grip strength and lack of help with fine activities, and one citing social stigma concerns.
2021, Binedell,^38^ Singapore, Case report	Design and develop comfortable 3D-printed cosmetic forequarter prosthetic: lightweight, cool, elbow lock, matched arm, metalfree	N = 1, Age 60, Male, Forequarter, Trauma	Multi Jet Fusion, Polyamide, Handheld scanner, Compared to conventional device	QUEST 2.0	The 3D-printed forequarter prosthesis was strongly preferred for overall effectiveness, accurate sizing, and ease of use/suspension, with total QUEST scores improving from 4.13 to 4.75. Scores improved in nearly all categories except durability (which decreased) and comfort (remained stable at 4). Concerns were raised about long-term durability, the elbow locking mechanism, and the aesthetic appearance of the mesh structure.
2021, O'Brien,^37^ Australia, Case report	Compare grip strength, dexterity, and consumer perception for two novel 3D-printed devices with a conventional myoelectric prosthesis	N = 1, Age late 20s, Male, Digit/partial hand, Undefined etiology	FDM, ABS, Undefined scanning, Compared to conventional device	Box and Block Test, Grip/pinch strength, Consumer Prosthesis Use and Satisfaction Questionnaire	Both 3D-printed devices demonstrated very poor grip and pinch strength but achieved comparable dexterity to the myoelectric prosthesis on the BBT. The patient reported higher satisfaction with 3D-printed devices for weight and thermal attributes, but the myoelectric prosthesis was rated superior for fit, grasping ability, overall function, ease of control, and general satisfaction.
2021, Ratto,^62^ Uganda/Tanzania/Cambodia, Non-randomized experimental study	Compare the efficacy of 3D-printed and conventional transtibial prostheses in large patient populations in low-income countries over time	N = 61, Age 5–25, 36 Male, 25 Female, Transtibial, Undefined etiology	FDM, Polyamide, Handheld scanner, Compared to conventional device	Prosthesis Evaluation Questionnaire, Device condition assessment	Conventional prostheses were rated significantly higher for functional mobility (walking, stairs, hills, slippery surfaces), duration of use, fit, comfort, and texture. 3D-printed prostheses rated better only for sweat management. There were no significant differences in satisfaction, school/work attendance, or experiences of public ridicule. 3D-printed devices showed higher rates of structural issues including loose fit (11.48% vs 6.56%), minor damage (3.29% vs 0%), and complete breakage (6.56% vs 0%).
2021, Sterkenburg,^49^ Sierra Leone, Case series	Investigate impact of 3D-printed prosthesis on health-related quality of life in upper extremity amputees in Sierra Leone Investigate short-term	N = 7, Mean age 40, 3 Male, 4 Female, digit/partial hand, transradial, transhumeral, proximal to shoulder, trauma/infection	FDM, PLA, Handheld scanner, No comparator	EQ-5D-5L, questionnaire	Overall health-related quality of life increased 18.8% across the cohort (mean EQ-5D-5L), with all patients showing improvement at first follow-up (mean 2.2 months). At second follow-up (mean 8.7 months), 5 patients reported improved HRQOL while 2 patients reported a decrease. Participants reported increased self-confidence, valued both aesthetics and function equally, and felt more included in society. Two patients experienced skin issues with prolonged wear, indicating need for material or fit refinement.
2021, van der Stelt,^56^ Sierra Leone, Case series	Investigate short-term feasibility and functionality of low-cost, locally produced 3D-printed transtibial prosthetic sockets in rural Sierra Leone using questionnaires to assess use, satisfaction, complications, and goal achievement	N = 8, Mean age 43 ± 15, 3 Male, 5 Female, Transtibial, Trauma/infecti on/ulcer	FDM, PLA, Handheld scanner, No comparator	Usage and satisfaction questionnaires	After 6 weeks, all participants continued wearing the prosthesis with 6 of 8 achieving their personal rehabilitation goals. All participants eliminated their need for crutches. Complications were minor (one pressure spot, one transient stump discomfort). Participants valued aesthetics equally with functionality. Note: Study title states observational cohort; classified here as a case series given short follow-up and absence of a defined comparison group.
2022, Copeland,^40^ USA, Case report	Compare functional outcomes and patient satisfaction between standard transradial prosthesis fitted in clinic and a 3D-printed prosthesis fitted remotely	N = 1, Age 59, Male, Transradial, Trauma	FDM, PLA, Photography, Compared to conventional device	Box and Block Test, Bimanual Coordination Tray Test, OPUS, QUEST 2.0	The remotely fitted 3D-printed prosthesis demonstrated significantly better dexterity performance (BBT 17.0 vs 12.3 blocks) with no difference in bimanual coordination. The clinic-fitted conventional prosthesis achieved higher device satisfaction on QUEST (4.25 vs 3.25), while the 3D-printed scored higher on overall OPUS satisfaction measures (4.2 vs 3.6). The patient ultimately preferred the conventional prosthesis overall, though rated the 3D-printed device better for size, weight, wearability, and aesthetics. Concerns about durability and individual finger dexterity limited 3D-printed device acceptance.
2022, Lee,^51^ South Korea, Case series	Report of 2 patient cases with work-related finger amputation who received body-powered 3D-printed finger prostheses and training	N = 2, Age 25–26, 1 Male, 1 Female, Digit/partial hand, Trauma	FDM, ABS, No scanning, No comparator	Jebsen-Taylor Hand Function Test, Box and Block Test, Canadian Occupation Performance Measure, joint ROM, VAS Pain	Both patients experienced complete pain elimination (VAS 7 to 0 and 3 to 0) and showed functional improvements, though outcomes varied by duration of use. Patient 1 (3 months) showed substantial BBT improvement (33 to 49 blocks) and meaningful gains in patient-identified performance areas including writing, typing, and cooking (no change in JTHFT). Patient 2 (1 month) demonstrated improved JTHFT hand function test times and typing ability, though guitar playing remained unchanged.
2022, O'Brien,^58^ Australia, Pilot study	Investigate feasibility of 2-week real-world trial of the Self Grasping Hand (SGH), a novel 3D-printed passive adjustable hand prosthesis; measure wear time, function, satisfaction, and acceptability	N = 9, Age 19–62, 5 Male, 4 Female, Transradial, Trauma/infecti on/congenital	Undefined method, Undefined materials, Undefined scanning, Compared to conventional device	Wear time (accelerometer), OPUS, TAPES-R	Five participants used body-powered split-hooks, and four had passive cosmetic hands as their usual prosthesis. Cosmetic prosthesis users wore the SGH substantially more (250.57h vs 124.98h for their own device), while body-powered users wore it far less (52.57h vs 474.47h). Performance ratings were modest (OPUS 45.8–50/100) with moderate satisfaction (TAPES-R: aesthetics 2.17/3, function 2.07/3, overall satisfaction 5/10). All participants reported the release button was difficult to operate, and most (7/9) found the finger-thumb aperture too small for everyday objects. Body-powered users were unable to perform daily tasks with the SGH. Approximately one-third needed repairs during the trial, indicating durability concerns.
2023, Nickel,^59^ USA, Pilot study	Examine real-world effects of using 3D-printed sockets for veteran amputees in terms of mobility, comfort, balance confidence, and acceptance	N = 9, Age 45–70, Male, Transtibial, Undefined etiology	FDM, Composite (nylon reinforced with carbon fiber), Undefined scanning, Compared to conventional device	2-minute walk test, Socket Comfort Score, PEQ, PLUS-M, ABIS-R, ABC, Borg RPE	No significant differences were found between conventional and 3D-printed sockets across any outcome measures. Participants were generally satisfied with the fit of 3D-printed sockets. Technical challenges emerged with achieving elevated vacuum in 3D-printed sockets when using sealing sleeve and seal-in liner suspension methods, suggesting manufacturing refinements are needed for advanced suspension systems.
2023, Shrestha,^67^ Nepal, Cross-sectional study	Explore the functionality of the three dimension printed prosthetic hands using semi-structured questionnaires and assess a series of daily tasks after three months prosthetic use	N = 76, Age 5–18, 36 Male, 40 Female, Digit/partial hand and transradial, Trauma/conge nital/unspecified disease	FDM, PLA and PETG, No scanning, No comparator	Southampton Hand Assessment Protocol, Usage questionnaire	All 76 children achieved 100% success with basic tasks (lifting light objects, holding tennis balls/bottles, lifting trays) but showed moderate success with tasks requiring pinch grip or fine motor coordination (40–42% for picking coins/opening jars, 77.6% for pushing buttons) and 0% success with complex tasks requiring rotational force, heavy lifting, or cutting food. Despite functional limitations, 61.8% were completely satisfied with only 35.5% reporting complaints at 3 months. Device failures were common: 39.5% experienced rubber band tears, 15.8% cord breaks, and 9.2% prosthesis breakage.
2023, Thomas,^41^ USA, Case report	Evaluate if the application of a home exercise program utilizing a 3D-printed prosthetic hand resulted in increased range of motion, strength, coordination, sensory integration, and perceived function of the upper extremity	N = 1, Age 6, Male, Transradial, Congenital	Undefined method, Undefined materials, No scanning, No comparator	Bruininks-Oseretsky Test (BOT-2), ROM, Manual Muscle Testing, ABILHAND, Sensory Profile	The child demonstrated improvements across multiple physical domains including active wrist ROM (5–10° gains), passive forearm pronation, shoulder ROM in all directions, and muscle strength in most movements except radial deviation. Bilateral and overall upper-limb coordination improved on the BOT-2. Functional task performance improved for several ADLs (ABILHAND), with opening jars, toothpaste, unwrapping candy, and washing upper body becoming easiest.
2023, Trinler,^63^ Germany, Non-randomized experimental study	Compare biomechanical outcomes of people with a transtibial amputation using a novel, individualizable, 3D-printed prosthetic foot (ComfyStep, Mecuris) with two conventional, widely used prosthetic feet during level ground walking using a 3D motion analysis system	N = 10, Age 37–84, 7 Male, 3 Female, Transtibial, Undefined etiology	Selective Laser Sintering, Polyamide, No scanning, Compared to 2 conventional devices	Gait parameters/kine matics	Sound-side gait was comparable across all foot types. The affected side with the 3D-printed foot showed reduced ankle ROM (11.4° vs 16.4°/14.8° for conventional), increased plantar flexion moment, reduced power generation, larger roll-over radius, less favorable energy ratio (68.8% vs 86.3%/75.8%), and higher overall stiffness. Note: Study title states prospective cohort; however, we classified this as a non-randomized experimental study as there is within-subject comparison but no longitudinal follow-up.
2023, Yoshikawa,^52^ Japan, Case series	Develop the Finch, a lightweight 3D-printed prosthetic arm with three opposing fingers controlled by a myoelectric control system using the user's forearm muscle bulge	N = 5, Age 38–64, Male, Transradial, Trauma/conge nital	FDM, ABS, No scanning, Compared to conventional device	Southampton Hand Assessment Protocol	All participants completed abstract tasks in under 20 seconds on average (metal objects took longer than wood). Only two participants could complete ADL tasks with all tasks achieved except glass jug pouring. Participant B was able to complete the simulated ADL tasks with a conventionally produced i-limb, in which on average it outperformed the 3D-printed prosthesis in terms of task time completion and could pour the glass jug. The 3D-printed Finch achieved lower Index of Functionality scores (47 and 42) compared to the conventional i-limb (66). The device struggled with task requiring grasping force when fingers were nearly closed (food cutting, key rotating) due to low stiffness, and fingers unintentionally opened when handling heav objects.
2024, Eshraghi,^60^ Canada, Pilot study	Explore the feasibility of analyzing geometric and clinical differences between digitally- and manually-designed sockets	N = 9, Age not specified, Gender not specified, Transtibial, Undefined etiology	FDM, ABS, Handheld scanner, Compared to conventional device	Socket Comfort Score, Scanning/casting experience surveys	Socket comfort was comparable between manually-designed (8.3 ± 0.5) and digitally-designed sockets (7.8 ± 1.6) with no significant differences. Eight of nine patients were satisfied with their digitally-designed socket comfort. Unsatisfactory outcomes in one case resulted from inaccurate digital modifications by the clinician, including underestimating global reduction and improper modifications at the distal end.
2024, Phillips,^66^ Canada, Qualitative study	Provide an understanding of perceptions and experiences with digital technology for prosthetic socket manufacturing from the perspective of prosthesis users	N = 9, Age 35–70, 4 Male, 5 Female, Transtibial, Vascular disease/traum a/malignancy/i atrogenic	FDM, Undefined materials, Handheld scanner, No comparator	Socket Comfort Score, semi-structured interviews, qualitative thematic analysis	Six of nine participants (67%) accepted their 3D-printed socket; SCS scores were higher among those who accepted (8, 6.5, 8.5, 9, 9, 7.5) than those who rejected (4, 7, 5), for an overall average of 7.2/10. Two themes were identified: (1) expectations and prioritization of socket usability, where participants expected digital manufacturing to produce better-fitting sockets than conventional methods and were more disappointed when fit fell short, with fit and function outweighing the novelty of the technology itself; and (2) facilitators and barriers to uptake, where quick non-invasive scanning, clinician trust, and casting as a backup eased adoption while reduced material adjustability and loss of tactile feedback from hand-casting were barriers.
2025, Baumgartner,^53^ Austria, Case series	Construct and evaluate 3D-printed transradial socket for amputees to use within first month of amputation	N = 4, Age 30–45, Male, Transradial, Undefined etiology	FDM, Composite (micro carbon fiber-filled nylon), No scanning, No comparator	QUEST 2.0, Custom questionnaire	Overall satisfaction was moderate (3.92/5.0, “more or less” to “quite satisfied”) with highest ratings for breathability (4.69), ease of donning/doffing (4.39), and weight (4.31). Lowest satisfaction ratings were for dimensions (3.71), durability (3.58), and comfort (3.54) While all participants supported the concept for early clinical fitting and training, they preferred conventional sockets for long-term daily wear. Experts rated the socket more favorably than end users.
2025, Dimintiyanova,^65^ India, Qualitative study	Enhance understanding of 3D-printed prosthesis acceptability; explore how children utilized devices (mothers' perspectives)	N = 7 (mothers of children), Children age 3–12, Gender not specified, Digit/partial hand and transradial, Congenital	Undefined method, Undefined materials, No scanning, No comparator	Usage questionnaire, Semi-structured interviews, Qualitative themes	Mothers reported concerns including slippery plastic material affecting grasping, aesthetic concerns (device didn't match skin color), pain from wrist flexion during gripping, and insufficient grip strength limiting all functionality. Younger children experienced difficulty adapting and found no assistance with tasks already manageable without prosthesis, while older children struggled with heavier items. Most children discontinued use or used the device for limited duration due to perceived ineffectiveness and social stigma. One child with below-elbow CULD, was the exception.
2025, Joo,^43^ South Korea, Case report	Evaluate the clinical efficacy of a customized three-dimensional printed robotic finger prosthesis in a 24-year-old man who sustained left second finger loss due to electrical burns	N = 1, Age 24, Male, Digit/partial hand, Trauma	Selective Laser Sintering, Polyamide and thermoplastic polyurethane (TPU), Handheld scanner, No comparator	Jebsen-Taylor Hand Function Test, Grip strength, MHQ, OPUS, TAPES-R, Modified Barthel Index	The robotic finger prosthesis significantly increased grasp and pinch power, improved JTHFT hand function test performance (page turning, writing speed, object lifting), and enhanced daily activities (Modified Barthel Index increased). Psychosocial adjustment improved (TAPES-R), and upper limb functionality was significantly enhanced (OPUS). However, the patient reported mixed results on the MHQ with improved aesthetics, satisfaction, and work capability, but paradoxically decreased ratings for function and ADL performance.
2025, Romero,^54^ Peru, Case series	Design, develop, and evaluate an affordable, personalized transradial myoelectric prosthesis combining 3D printing with AIbased EMG control; demonstrate feasibility of localized digital manufacturing for prosthetic customization in low-resource settings	N = 9, Age 18–60, 5 Male, 4 Female, Transradial, Trauma/conge nital	FDM, PLA, Handheld scanner, No comparator	Brief Activity Measure for Upper Limb Amputees (BAM-ULA)	The AI-driven prosthesis achieved good overall functionality (average BAM-ULA 7.4/10) with 100% success for gross motor tasks but only 22.2% success for fine motor tasks. Users found the prostheses easy to use though some experienced minor discomfort from sweating after extended use. Muscle fatigue was highe among users without prior prosthesis experience, suggesting importance of training and conditioning. Limitations included lower success with precision grip and wrist articulation, and high energy consumption for the AI interface.
2025, Rossi,^42^ USA, Case report	Examine impact of 3D-printed prosthetic hand on function and quality of life	N = 1, Age 13, Male, Digit/partial hand (difference, having a palm without digits and a decreasedsized thumb), Congenital	FDM, PLA, Photographs, No comparator	OPUS (UEFS and HRQOL surveys)	Functional status improved meaningfully (UEFS 31 to 50) with improvements in 4 specific activities (drinking from cup, pouring can, carrying laundry, applying toothpaste), though 19 tasks remained incomplete with the prosthesis. Health-related quality of life showed minimal change (HRQOL 58 to 61, below minimal detectable change) with mixed results: decreased social withdrawal and higher physical accomplishment, but increased pain and feelings of being downhearted/depressed.
2025, Silva,^26^ Portugal, Case report	Propose and demonstrate a clientcentered methodology for designing and producing 3D-printed assistive devices for children with congenital limb defects; present a case example of multifunctional assistive device for a 7-year-old child	N = 1, Age 7, Male, Transradial (hypoplastic forearm), Congenital	FDM, PLA, No scanning, No comparator	System Usability Scale, Feeding and guitar pick testing	The child successfully demonstrated independent performance with a knife and playing guitar after brief training, achieving high usability ratings (SUS 4-5 for positive items, 1-2 for negative items) with no reported discomfort. The multi-activity device enabled two previously impossible activities, demonstrating meaningful functional gains. Limitations included difficulty assembling the device independently and PLA material susceptibility to deformation in high heat.
2025, van der Stelt,^61^ Sierra Leone, Longitudinal cohort study	Evaluate the durability and usage of 3D-printed transtibial prosthetic sockets, as well as to assess the impact of these prostheses on participants’ functioning and health-related quality of life (HRQoL)	N = 9, Age 23–62, 6 Male, 3 Female, Transtibial, Vascular disease/traum a/infection/sna ke bite	FDM, PLA, Handheld scanner, No comparator	Step count, wear time (accelerometer), OPUS (LEFS and D-HRQoL, adapted), EQ-5D-3L, VAS	At 2.5-year follow-up (n = 6), participants maintained high device use (median 12.4 hours/day, 6,906 steps/day with 65% of steps taken with prosthesis). Functional status showed modest improvement trends (OPUS LEFS 47 to 55, p = 0.063), while diseasespecific quality of life (OPUS D-HRQoL 17/36 to 34/36, p = 0.018), general quality of life (EQ-5D-3L 0.741 to 0.854, p = 0.018), and self-perceived health (VAS 6/10 to 10/10, p = 0.027) improved, albeit below the study’s statistical significance threshold of p = 0.01. Structural durability concerns emerged with socket damage in 6 cases over 2.5 years (primarily patella region breaks). Qualitatively, some participants reported meaningful functional gains despite minimal score improvements.
2025, Wiesner-Luna,^55^ Colombia, Case series	Explore the functionality and everyday uses of a low-cost, 3D-printed body-powered upper limb prosthesis for children/adolescents with congenital limb absences	N = 4, Age 4–13, 2 Male, 2 Female, Transradial, Congenital	FDM, PLA and NinjaFlex, No scanning, No comparator	Jebsen-Taylor Hand Function Test, UNB Test of Prosthetic Function	Performance varied by task complexity: fine motor tasks showed delays exceeding 30 seconds compared to non-prosthetic limb performance, while gross motor tasks demonstrated delays under 30 seconds. Three of four participants showed moderate spontaneity and ability scores (2/4 for spontaneity, 2–3/4 for ability), with better performance in gross motor activities (carrying, opening containers) than fine motor tasks requiring bilateral coordination. Parents reported meaningful improvements in ADL participation, school activities, and psychosocial outcomes including self-confidence, selfesteem, and body image. However, absence of formal training led to inconsistent spontaneous use, frustration when functional expectations were unmet, and some discontinuation. None of the participants received preprosthetic training.

## Supplementary Digital Content 1: Search Strategy

A comprehensive search strategy was developed to identify relevant studies on digitally manufactured prosthetic and orthotic devices, focusing on clinical outcomes. The search strategy combines controlled vocabulary (MeSH terms) and free-text keywords, ensuring broad coverage of relevant literature.

**Reproducible search strategy for Ovid Medline**

1. exp Amputation, Surgical/
2. Amputation Stumps/
3. exp Amputation, Traumatic/
4. Amputees/
5. exp Limb Deformities, Congenital/
6. amput*.mp.
7. disarticulat*.mp.
8. ((ankle* or arm or arms or elbow* or finger* or feet or foot or hand or hands or hip* or knee* or leg* or limb* or lower extremit* or shoulder* or thumb* or upper extremit* or wrist*) adj5 (deficienc* or deformit* or differenc* or loss or pathol* or reduc* or residual)).mp.
9. hemipelvectom*.mp.
10. phocomel*.mp.
11. prosthe*.ab./freq=3
12. or/1-11 [population - people with amputation/limb difference]
13. Artificial limbs/
14. Orthotic Devices/or Braces/or Foot Orthoses/
15. ((artificial or orthotic* or orthos?s or prosthe*) adj5 (ankle* or arm or arms or elbow* or finger* or feet or foot or hand or hands or hip* or knee* or leg* or limb* or lower extremit* or shoulder* or thumb* or upper extremit* or wrist*)).mp.
16. or/13-15 [prosthetic and orthotic devices concept]
17. exp Computer-Aided Design/
18. ((3-d or 3d or additive or computer-aided or digital* or rapid or three-d or three-dimensional) adj5 (fabricat* or manufactur* or print* or prototyp*)).mp.
19. cad-cam.mp.
20. computer-aided design*.mp.
21. fused deposition model*.mp.
22. laser sinter*.mp.
23. (multijet fusion or multi-jet fusion).mp.
24. (stereolithograph* or stereo-lithograph*).mp.
25. or/17–24 [computer-aided design concept]
26. and/12,16,25

**Filters and Limits:**

- English language only
- Peer-reviewed journal articles
- Date restrictions: Up to December 31, 2025

Search conducted in MEDLINE (Ovid) on January 16, 2026. The search was adapted for other databases by modifying subject headings and syntax accordingly.

## Abbreviations

**3D-printing techniques**

- FDM – Fused Deposition Modeling
- SLS – Selective Laser Sintering
- MJF – Multi Jet Fusion

**3D-printing materials**

- PLA – Polylactic Acid
- ABS – Acrylonitrile Butadiene Styrene
- PLACTIVE – Antibacterial PLA filament
- PETG – Polyethylene Terephthalate Glycol
- TPU – Thermoplastic Polyurethane (e.g., NinjaFlex)
- Polyamide – Polymer containing repeating amide bonds, commonly known as Nylon
- Composite – Composite filament (e.g., micro carbon fiber-filled nylon)

**Outcome measures**

- JTHFT – Jebsen-Taylor Hand Function Test
- BBT – Box and Block Test
- UNB test – University of New Brunswick Test of Prosthetic Function
- UBET – Unilateral Below Elbow Test
- ABILHAND – ABILHAND questionnaire for manual ability in daily activities
- SHAP – Southampton Hand Assessment Protocol
- BOT-2 – Bruininks-Oseretsky Test of Motor Proficiency, 2nd edition
- MHQ – Michigan Hand Outcomes Questionnaire
- 2MWT – Two-Minute Walk Test

**Questionnaires**

- OPUS – Orthotics and Prosthetics Users' Survey
- UEFS – Upper Extremity Functional Status (OPUS subscale)
- LEFS – Lower Extremity Functional Status (OPUS subscale)
- QUEST 2.0 – Quebec User Evaluation of Satisfaction with Assistive Technology, version 2.0
- PEQ – Prosthesis Evaluation Questionnaire
- TAPES-R – Trinity Amputation and Prosthesis Experience Scales – Revised
- SCS – Socket Comfort Score
- PLUS-M – Prosthetic Limb Users Survey of Mobility
- ABIS-R – Amputee Body Image Scale–Revised
- CAPP – Child Amputee Prosthetics Project
- SUS – System Usability Scale
- PSFS – Patient-Specific Functional Scale
- VAS – Visual Analog Scale
- ABC – Activities-specific Balance Confidence Scale
- RPE – Borg Rating of Perceived Exertion
- EQ-5D-5L – EuroQol 5-Dimension, 5-level questionnaire
- EQ-5D-3LEuroQol 5-Dimension, 3-level questionnaire

**Other**

- ADL – Activities of Daily Living
